# Open questions on atmospheric nanoparticle growth

**DOI:** 10.1038/s42004-020-00339-4

**Published:** 2020-08-07

**Authors:** Taina Yli-Juuti, Claudia Mohr, Ilona Riipinen

**Affiliations:** 1 Department of Applied Physics, University of Eastern Finland, Kuopio, Finland; 2 Department of Environmental Science (ACES), Stockholm University, Stockholm, Sweden

**Keywords:** Atmospheric chemistry, Organic chemistry

## Abstract

Cloud droplets form in the atmosphere on aerosol particles, many of which result from nucleation of vapors. Here the authors comment on current knowledge and open questions regarding the condensational growth of nucleated particles to sizes where they influence cloud formation.

## Particle growth is relevant for the climate impact of particles formed in the atmosphere

**Fig. 1:**
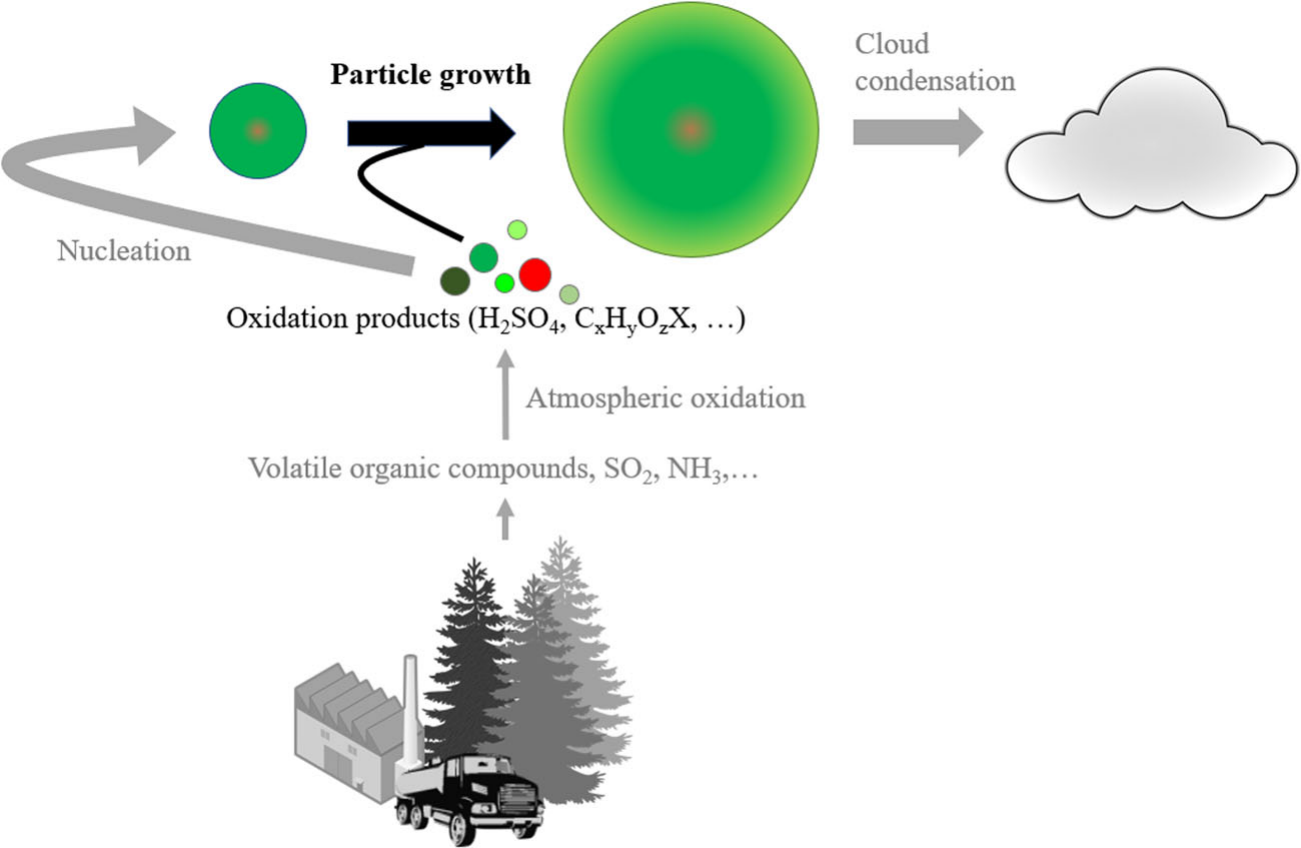
Nanoparticle growth by vapors to sizes relevant for cloud formation. Volatile emissions from biogenic and anthropogenic sources form condensable vapors in the atmosphere through oxidation. Some of the oxidized vapors form new particles via nucleation and/or grow the formed particles towards climatically relevant sizes. Colored parts in the schematic indicate the focus of this Comment article. Parts of the figure are from publicdomainvectors.org and used under the Creative Commons Deed CC0.

Secondary organic aerosol (SOA) contributes significantly to atmospheric fine particle mass. The SOA formation process includes emissions of volatile hydrocarbons, their multi-step oxidation in the atmosphere, and partitioning of the oxidation products into the condensed phase leading to particle growth^1^ (Fig. 1). Processes limiting condensational growth vary with particle size. A somewhat special aspect of SOA formation is new particle formation (NPF) via nucleation^2^. Here we comment on current knowledge and open questions regarding the condensational growth of nucleated particles from a few nanometers, to sizes where they can impact the climate, and give recommendations for future research. We focus on the growth by organic compounds where important questions are yet to be answered despite of the progress over the last decades.

A substantial fraction of particles acting as cloud seeds (called cloud condensation nuclei, CCN) originate from nucleation^3^. Particle growth plays a critically important role here: to act as CCN, the formed tiny particles usually need to reach tens of nanometers (nm) in diameter. Growth competes with particle loss through coagulation—the faster the particles grow, the larger the fraction that survive to CCN size. An order-of-magnitude growth in diameter from e.g., 5 to 50 nm increases the particle volume by three orders of magnitude. The composition of the particles is therefore largely defined by the compounds that grow them by condensation, often organic vapors. Comprehension of particle growth and its reasonable description in global and regional scale models is needed to improve our understanding of aerosol-cloud interactions^4^, which are one of the most uncertain aspects of anthropogenic climate forcing^5^.

## Particle growth rates and the vapors growing the particles

Particle size distribution observations and diameter growth rates derived from them are essential for understanding the growth of freshly formed nanoparticles. Measurements from various environments^6^ show particle growth rates of the order of 1–10 nm/h. Currently available data provide information of variability and seasonality of nanoparticle growth, although the majority of the observations are from the Northern hemisphere, specifically from ground-based remote or rural continental sites in Europe.

**Fig. 2:**
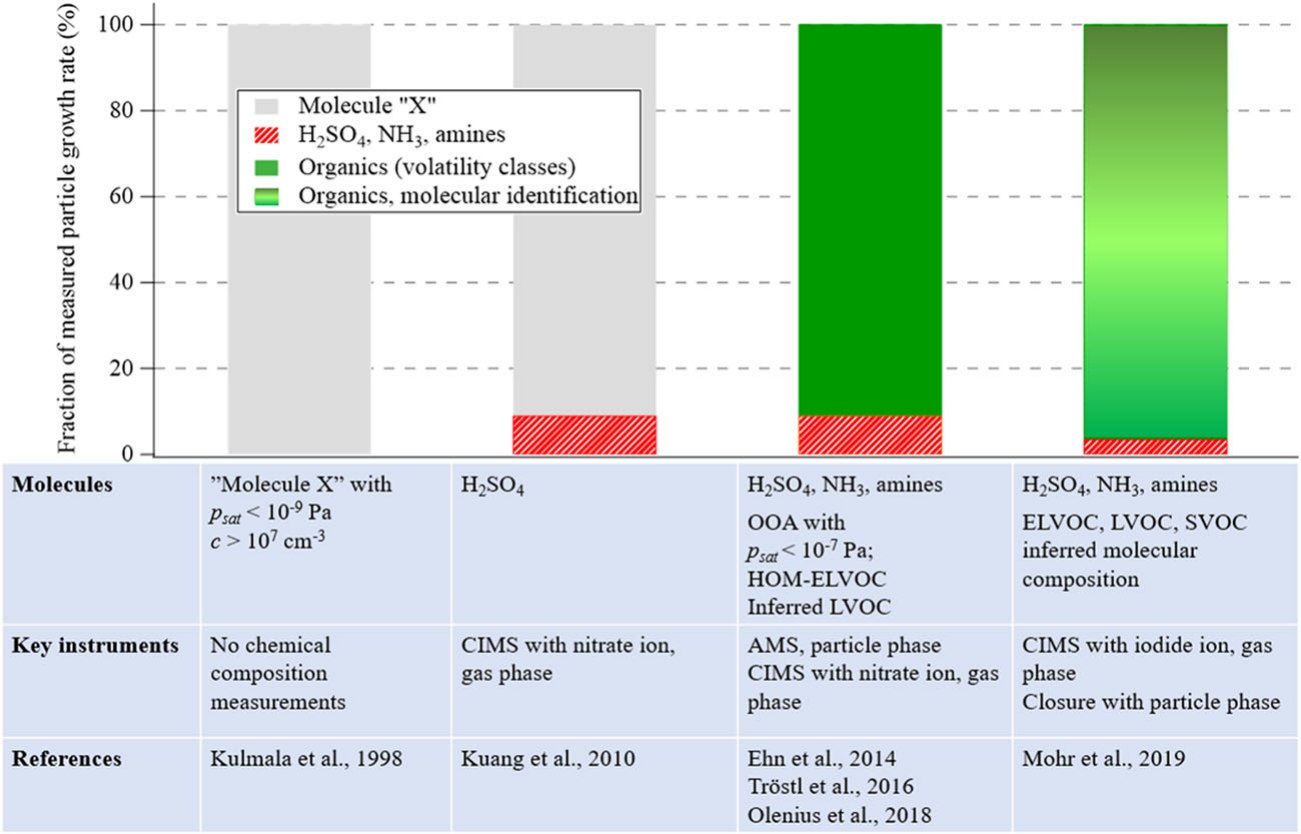
Evolution of understanding of nanoparticle growth in boreal forests. Various evidence has contributed to building the picture of the significant role of organic vapors in nanoparticle growth in the boreal forest. Initially, frequent new particle formation and growth was observed without identification of the condensing vapors (Kulmala et al.^17^). Later, sulfuric acid was found to explain a fraction of the growth (e.g., Kuang et al.^18^), and direct and indirect evidence led to understanding of the significant role of organics (shown by e.g., Ehn et al^7^. and reviewed in Olenius et al.^2^) which was supported by laboratory simulations (Tröstl et al.^8^). Recently, it was shown that the detected organic vapors—accounting for their volatilities—could explain the observed nanoparticle growth at a boreal forest site with a small contribution of sulfuric acid (Mohr et al.^10^). *p*_*sat*_ = saturation vapor pressure, *c* = gas phase concentration, AMS = Aerosol Mass Spectrometer, CIMS = Chemical Ionization Mass Spectrometer, OOA = oxygenated organic aerosol, HOM = Highly oxygenated organic molecule.

Knowledge about the properties of the vapors that grow the particles has increased but is still incomplete (Fig. 2). Sulfuric acid, which plays a key role in nucleation, and other inorganic compounds such as ammonia, contribute to condensational growth in many environments. However, it has become evident that organic vapors are responsible for most of the particle growth in environments with a source of organic precursor gases^2^. The advancement of mass spectrometric techniques in the last few years has enabled the identification of source groups and compounds contributing to particle growth, for example highly oxygenated organic molecules^7–10^. However, their multi-phase nature and complexity have made the detection of individual organic molecules and their contributions to nanoparticle growth challenging. Given the fundamental role volatility plays in evaporation and condensation, one simplification is grouping condensable organics into experimentally determined volatility classes: extremely low, low, semi, and intermediate volatile organic compounds abbreviated as ELVOC, LVOC, SVOC, and IVOC, respectively. There is general understanding that the smaller the particles, the lower the volatility of a compound has to be in order for it to condense. In other words, the larger the particles, the larger the contribution of SVOC to their growth.

In the absence of particle-phase chemistry or transport limitations, condensation of known compounds to homogeneous liquid droplets is well described by basic mass transport models^2^. Hand-in-hand with the increasing amount of experimental data, also the availability and robustness of different predictive models for the thermodynamics of atmospherically relevant compounds and mixtures has increased^11^. These steps forward have allowed for the development of models describing the growth and evaporation of atmospheric >5 nm particles with good accuracy if the concentrations and molecular properties of the vapor phase compounds are known^9,10^. We have recently been able to capture nanoparticle growth in the boreal forest, and to characterize the organic vapors involved, by using a combination of chemical ionization mass spectrometry and a condensation model^10^. Therein we confirm the crucial role of oxidation products of biogenic volatile organic compounds for particle growth in this environment. Also on global and regional scales, the inclusion of a description of nanoparticle growth by organic compounds was shown to improve predictions of aerosol size distributions, hence suggesting that this process is important for capturing e.g., aerosol impacts on clouds^4,12^.

Nanoparticle growth spans different phases, thermodynamic and kinetic regimes, and spatial and temporal scales. Further scientific progress on this topic requires a multidisciplinary and multiangle approach. In the following we will discuss a few examples of open research questions and ongoing research efforts.

## Open questions and ongoing research efforts regarding nanoparticle growth

To date, a consistent picture for the role of organic precursors other than monoterpenes, particularly α-pinene, is still developing. Particle growth mechanisms in environments other than the boreal forests, for example in the marine boundary layer, are not well understood. Ongoing research efforts concentrate for instance on isoprene, the most abundant VOC, and its oxidation products. Isoprene suppresses NPF and lowers SOA yields in mixed precursor environments^13^. There is indication that isoprene oxidation products may contribute to the high particle numbers observed in the upper troposphere over the Amazon^14^. At such high altitudes the lower temperature decreases the volatility of the molecules and shifts the equilibrium towards the particle phase. With the recent elucidation of the importance of NPF in urban environments^15^, we expect progress on understanding particle growth in polluted environments to be imminent, despite the increased complexity of the mix of anthropogenic and natural precursor gases and oxidants.

Compared to gas or nanoparticle composition observations, growth rate measurements are more common and considered more robust. However, the interpretation of point observations of a particle population passing a measurement site in terms of growth is challenging due to possible effects of changes in air mass or vertical boundary layer mixing. In addition, traditional methods for calculating the growth rate based on mode peak diameter may not provide pure condensational growth rates when coagulation is significant. Also, these methods are fundamentally limited by the time resolution of the instrumentation. Development of promising new methods based on particle population dynamics is ongoing^2^.

Net particle growth is a competition between condensation and evaporation. Whereas the former is driven by the concentration of the condensing vapors, the volatilities of the condensed compounds govern the latter. Pure compound saturation vapor concentrations C_sat_ are therefore needed for accurate predictions of particle growth. However, the complex mixtures formed in the growing particles may not behave ideally and particle composition dependent activities need to be accounted for. There are large uncertainties how particle-phase chemical reactions or the formation of a viscous phase^2^ influence particle-phase volatilities, diffusivity and nanoparticle growth. This has led to e.g., the use of mass accommodation coefficients below unity for organic molecules colliding on SOA particles. We advise against below unity values due to the different ways diffusivity limitations or other SOA properties affect particle growth dynamics.

Experimentally determining C_sat_ of all oxidized organic compounds in the atmosphere is nearly impossible^11^. Hence, parametrizations of C_sat_, or the effective vapor concentration C* including non-ideality effects, with e.g., the molecular composition as input^16^ are increasingly used to obtain estimates of organic volatility distributions. However, the lack of molecular structure information, and again, far-from-complete representation of the complexity of the range of atmospheric compounds in developing the parametrizations, results in orders-of-magnitude uncertainties in saturation vapor concentrations^10^. As the experimental techniques for identifying molecular composition of oxidized compounds are becoming more common, it is crucial to develop more precise methods to convert the molecular compositions into volatility information.

## Outlook

Ultimately, an increased understanding at the molecular process level should lead to an improved description of the climate system at the global level. Assessing the climate impact of nanoparticle formation and growth therefore depends also crucially on our ability to represent them in large-scale models. Besides improving the fundamental understanding of the properties of the condensing species, it is also worthwhile to systematically investigate the minimum number of variables needed to represent their effects on the evolution of aerosol size distribution and composition. The optimal compromise between representing the complex particle growth processes and computational demands depends on the question at hand.

A few words on particle growth below 5 nm: At these sizes, the standard theoretical approaches applied for condensation and evaporation are not applicable anymore^2^. Together with experimental challenges related to sampling and detection of the vapor and cluster populations, these issues make the interpretation of sub-5 nm particle growth observations challenging. Further work in the development of molecular resolution models and their systematic upscaling for various atmospherically relevant systems are therefore needed^2,8^.

We expect the steep instrumental progress to continue in the next decade, and higher time- and chemical resolution data will become more widely available from various environments and different systems. The ability to process this wealth of data will likely become critical for improving our understanding on SOA, and emerging techniques based on e.g., machine learning will be needed. To support such analysis and the building of comprehensive understanding of the organic molecules in various environments, establishing open databases that combine the complex data and allow for broad use of the accumulating data will be helpful. We expect an increasing number of large-scale models to implement and/or improve the volatility representation for the condensing organic molecules in the coming decade, e.g., with a VBS (volatility basis set) scheme. This will allow for better assessment of the climate impacts of the atmospheric NPF, including nanoparticle growth.
